# Impact of Sex on Infection Risk in Patients with Systemic Lupus Erythematosus

**DOI:** 10.3390/bioengineering12010059

**Published:** 2025-01-13

**Authors:** R. Borrelli, S. Nicola, F. Corradi, I. Badiu, L. Lo Sardo, N. Rashidy, A. Quinternetto, M. Mazzola, F. Meli, E. Saracco, I. Vitali, S. Negrini, L. Brussino

**Affiliations:** Allergy and Immunology Unit, Department of Medical Science, University of Turin, Mauriziano Hospital, 10128 Turin, Italy

**Keywords:** lupus, systemic lupus erythematosus, SLE, infections, sex

## Abstract

**Background:** Systemic lupus erythematosus (SLE) is a chronic autoimmune disease that exhibits considerable diversity in terms of both clinical and immunological manifestations. Since its female-to-male ratio is around 9:1, it is well recognized that systemic lupus erythematosus mostly affects women, especially those of childbearing age. There is a greater susceptibility to infections in adult patients with systemic lupus erythematosus (SLE) compared to the general population. However, only a small number of studies have attempted to analyze this risk using real-life data, and even fewer have successfully assessed the influence of sex. **Materials and Methods:** A retrospective study was conducted, enrolling patients and dividing them into two groups based on their biological sex. Infectious episodes were identified from medical records and categorized by severity. Patients were stratified according to disease duration and treatment received. Logistic regression analysis was used to calculate the odds ratio (OR), with a 95% confidence interval (CI) for the assessment of risk factors. Multivariable logistic regression was performed to adjust for potential confounders. Model fit was evaluated using the Hosmer–Lemeshow test, and interactions between variables were tested. Sensitivity analyses were conducted to assess the robustness of the findings. **Results:** A total of 119 patients (107 females and 12 males) were included in the analysis. No significant difference in age was found between sexes (t = −0.715, *p* = 0.487), but disease duration was significantly shorter in males (t = 3.35, *p* = 0.003). Logistic regression showed a significant association between male sex and infection risk (β = 0.9426, *p* = 0.05), with males having an almost sixfold higher probability of infection compared to females (OR 5.675, 95% CI: 1.4479–22.2477, *p* = 0.0127). Disease duration (β = 0.0250, *p* = 0.102) and smoking status (β = 0.4529, *p* = 0.078) were not statistically significant. Lastly, correlation analysis revealed a significant association between SS-A antibodies and infection rate (r = 0.291, *p* = 0.003). **Conclusions:** This study highlights a significant sex-based disparity in the risk of infections among SLE patients, with males being at a higher risk compared to females. The differences in the distribution of infections, such as the higher prevalence of pneumonia in males and urinary tract infections in females, suggest that sex-specific factors, including immunological and hormonal differences, may influence infection susceptibility. Our findings emphasize the need for tailored clinical management, with increased vigilance for infections in male patients, to improve prevention strategies and targeted therapeutic interventions in this subgroup.

## 1. Introduction

### 1.1. Background

Systemic lupus erythematosus (SLE) is a chronic autoimmune disorder characterized by a wide variety in both clinical and immunological manifestations [1].

Compared to the general population, patients with SLE are more likely to develop infectious events [2]. In fact, at least 50% of individuals encounter a serious infection, as they account for 11–23% of hospitalizations. To date, bacterial infections are the predominant causative factor in SLE patients (51.9%), with viruses accounting for 11.9% and lastly fungi for 2.3% [3].

SLE predominantly affects women, particularly during their reproductive years, with a female-to-male ratio of approximately 9:1 [4]. However, notable differences exist between male and female SLE patients. Males tend to present with more severe forms of the disease and are generally diagnosed at an older age, often up to six years later than females [5]. Clinically, male patients are more prone to severe manifestations, including significant skin lesions, serositis, thrombotic events, and neurological complications. Renal involvement is more common in males, with an increased susceptibility to lupus nephritis, frequently progressing to end-stage renal disease [6,7].

### 1.2. Sex Hormone-Induced Variations in Immune Response

Differences in SLE across sexes are influenced by genetic, hormonal, and immune response variations. The function of estrogen and its receptors in the pathogenesis is substantial, since they enhance immunological responses that worsen the disease, whereas androgens tend to improve it [6].

Sex-specific differences in Toll-like receptor (TLR) expression and TLR-driven interferon-alpha (IFN-α) production have been identified in the sexes. TLRs, which are pattern recognition receptors essential for innate immunity, can contribute to autoimmune diseases when their responses are dysregulated. In SLE, intracellular TLR7, TLR8, and TLR9 in plasmacytoid dendritic cells recognize nucleic acids, triggering type I interferon production, a key mediator in SLE pathogenesis characterized by elevated IFN-α levels and an increased expression of interferon-inducible genes.

TLR7 and TLR8, encoded by adjacent genes on the X chromosome, can escape X-chromosome inactivation (XCI) in women, leading to increased TLR expression and enhanced IFN-α production by B cells in response to stimulation. This sex-specific IFN-α production pathway appears independent of estrogen signaling for TLR7, although TLR8 expression requires estrogen receptor alpha (ERα) binding to an estrogen-responsive element near the TLR8 gene locus. Estradiol-bound ERα may further enhance TLR8 expression by activating signal transducer and activator of transcription 1 (STAT1). Additionally, TLR4-mediated cytokine responses, such as TNF-α, IL-1β, IL-6, and IL-8, fluctuate with the menstrual cycle, suggesting sex hormone influence, although the exact mechanisms remain unclear [8,9,10].

Other factors concur with the sex-based differences observed in SLE patients.

The molecular mechanisms of miRNA expression exhibit notable differences between males and females, particularly influencing autoimmune conditions like SLE. miRNAs, small non-coding RNAs that regulate gene expression post-transcriptionally, show sex-specific expression patterns in various tissues, including the brain, immune cells, and gonads. These differences are partly due to the genetic contribution of X-linked miRNAs, with about 113 miRNAs located on the X chromosome compared to only two on the Y chromosome. Such a genetic disparity contributes to sex differences in immune function and susceptibility to autoimmune diseases [6,11].

Estrogen plays a significant role in modulating miRNA expression, influencing immune responses differently in males and females. In females, estrogen affects miRNA biogenesis and function through estrogen receptors, altering the expression of miRNAs involved in immune regulation. For instance, estrogen upregulates miR-223 and miR-18a while downregulating miR-146a in splenocytes, which modulates inflammatory responses and type I interferon signaling pathways that are crucial in SLE. These estrogen-mediated changes in miRNA expression contribute to the heightened immune reactivity observed in females, enhancing susceptibility to autoimmune diseases. Additionally, estrogen can directly interact with miRNA biogenesis machinery, altering the processing of pri-miRNAs and impacting their maturation into functional miRNAs [8].

Sexual dimorphism in miRNA expression is also linked to the differential regulation of immune genes, where miRNAs such as miR-182, miR-31, and miR-155 show distinct expression patterns in female lupus-prone mice compared to males. These miRNAs contribute to the dysregulation of T and B cells, enhancing the autoimmune response in females [12,13].

Together, these molecular mechanisms highlight the intricate interplay between sex hormones, genetic factors, and miRNA regulation in shaping the sex-specific immune responses observed in SLE and other autoimmune conditions.

### 1.3. What This Work Adds

This study used real-life data to explore the sex differences in infection risk among SLE patients, distinguishing itself by using a comprehensive approach that accounts for confounding factors like disease duration and treatment. Unlike previous studies that merely focused on disease severity and activity, our research specifically highlights the distinct infection risk profile in males, offering a nuanced understanding of how sex influences infection susceptibility in SLE beyond the established clinical manifestations.

## 2. Materials and Methods

### 2.1. Study Design and Population

Outpatients with a verified diagnosis of SLE according to the 2019 American College of Rheumatology/European Alliance of Associations for Rheumatology (ACR/EULAR) classification criteria [13] were included in this retrospective monocentric study.

Infectious episodes were identified from medical records between 2017 and 2023 and categorized by severity.

The exclusion of patients with insufficient or partial medical documentation was implemented to guarantee the integrity of the data. Furthermore, we implemented other exclusion criteria, such as current neoplasia, the diagnosis of primary or secondary immunodeficiency, and substance addiction.

Records of current and previous therapies, comorbidities and immunosuppressants were also gathered for the analysis.

### 2.2. Objectives

The primary aim is to assess the role of sex in the risk of infection in patients affected by systemic lupus erythematosus and to assess for possible patterns or correlations between clinical factors.

### 2.3. Data Collection

Sex, age, age at diagnosis, smoking habits, ongoing and previous treatments, and infectious events were collected from medical records. Moreover, data on the patients’ serological profiles were also verified to determine possible correlations (anti-double-stranded DNA antibodies—anti-dsDNA—evaluated on fluorescent enzyme immunoassays—FEIAs—by ThermoFisher, Waltham, MA, USA and IFA on Crithidia luciliae-Euroimmun S.r.L. (Lübeck, Germany) for positive findings in order to confirm the result), anti-Ro/SS-A antibodies (divided, where available, into 52 kDa and 60 kDa, evaluated on fluorescent enzyme immunoassays—FEIAs—by ThermoFisher), and anti-La/SS-B and anti-Smith (anti-Sm, FEIAs byThermoFisher) antibodies.

Lastly, complement levels, namely C3 and C4, were recorded throughout the medical records.

### 2.4. Statistical Analysis

An analysis of the collected data was conducted using STATA SE 18.0 (1985–2023 StataCorp LLC, College Station, TX, USA).

The Shapiro–Wilk test was used to assess the normality of the data distribution to ensure appropriate model assumptions. Logistic regression analysis was used to calculate the odds ratio (OR) and 95% confidence interval (CI) for the assessment of risk factors.

Mann–Whitney U was used to evaluate differences in treatments and dosages among the groups. Multivariable logistic regression was performed to adjust for potential confounders. Model fit was evaluated using the Hosmer–Lemeshow test, and interactions between variables were tested. Sensitivity analyses were conducted to assess the robustness of the findings.

### 2.5. Ethical Approval

After receiving comprehensive information, all of the participants provided their consent. All the procedures were conducted in compliance with the relevant regulations, in line with the 1964 Declaration of Helsinki, and in compliance with the limitations established by the legislative body. The local Ethics Committee (Comitato Etico Territoriale interaziendale AOU Città della Salute e della Scienza di Torino, study #0116953) granted formal approval for this research.

## 3. Results

A total of 119 patients were included in the analysis (107 F, 12 M, F:M 8.92:1); the demographics are shown in Table 1 and comorbidities in Table 2.

Statistical analysis showed no significant difference in age between sexes (t = −0.715, *p* = 0.487), whereas disease duration was significantly shorter in males compared to females (t = 3.35, *p* = 0.003); The chi-squared test comparing disease severity (mild, moderate, or severe) between females and males revealed a weak, albeit statistically significant, difference (chi squared= 6.27, *p* = 0.043). As shown in Table 3 and Table 4 no difference in treatments nor in OCS dosages was observed.

**Figure 1:**
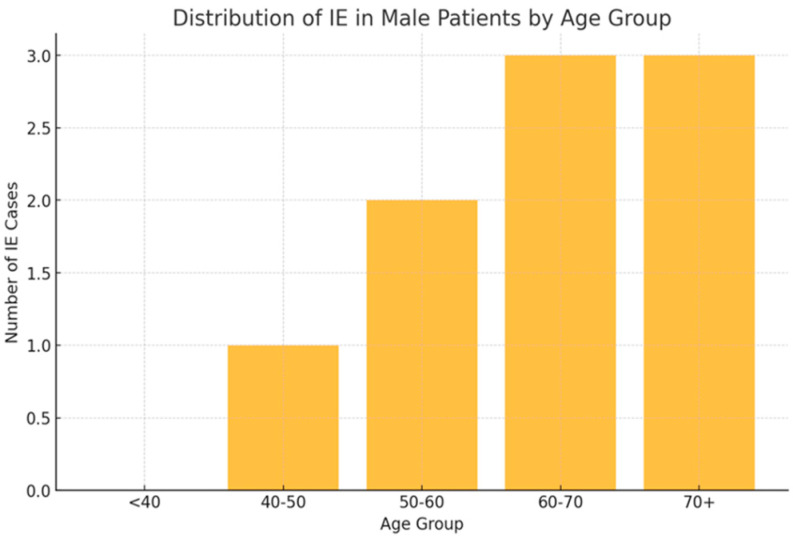
Distribution of infectious events (IEs) in males.

**Figure 2:**
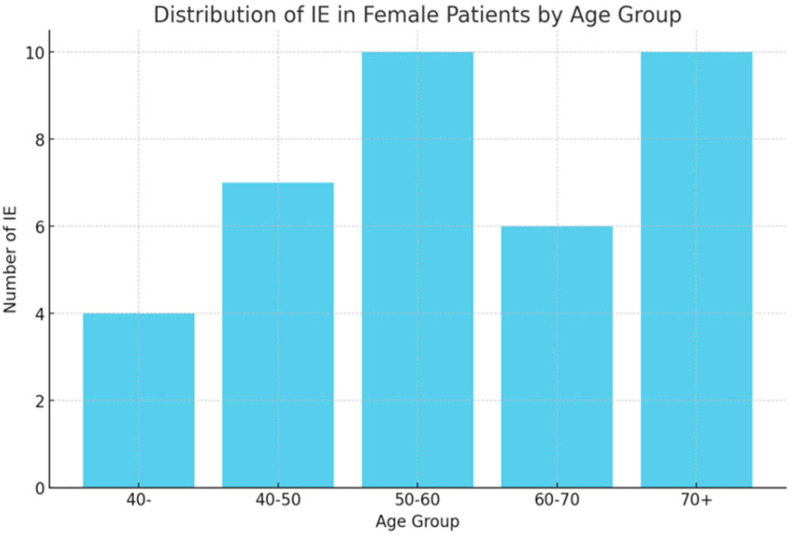
Distribution of infectious events (IEs) in females.

**Figure 3:**
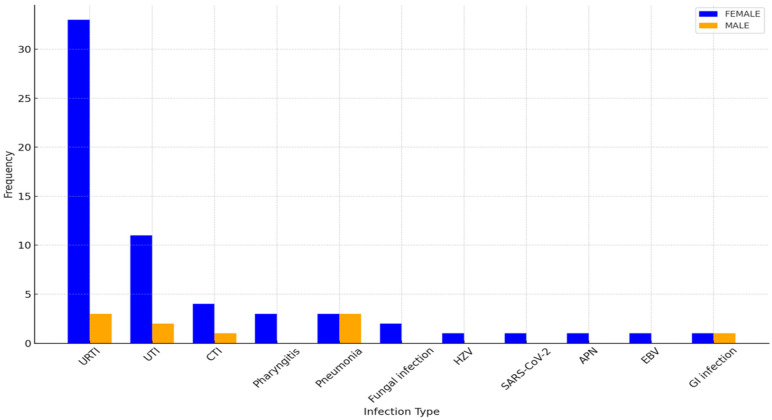
Distribution of infections for sex. URTI: upper respiratory tract infection, UTI: urinary tract infection, CTI: connective tissue infection, HZV: herpes zoster virus, SARS-CoV-2: severe acute respiratory syndrome coronavirus 2, APN: acute pyelonephritis, EBV: Epstein–Barr virus, GI: gastrointestinal.

Infectious episodes were categorized based on type and severity, with URTIs being the most prevalent in both sexes. As shown in Figure 1, Figure 2 and Figure 3, female patients exhibited higher incidences of URTIs (35.1%), followed by UTIs (21.6%), and lower respiratory tract infections (CTI) (10.8%). Among male patients, URTIs were also the most common (33.3%), followed by pneumonia (22.2%) and UTIs (22.2%).

## 4. Discussion

A logistic regression analysis was performed to assess the relationship between disease duration, sex, and smoking status with the risk of infections in patients with SLE.

Regarding sex, the logistic model indicated a statistically significant association between male sex and the risk of infections (β = 0.9426, Logit(*p*) = −0.6061 + 0.9426 × sex, *p*-value = 0.05).

A similar result was obtained via the odds ratio whilst comparing the infection risk between female and male patients as it showed an almost sixfold probability in the latter (OR 5.675, 95% CI: 1.4479; 22.2477, *p* = 0.0127).

On the other hand, disease duration (dd) failed to be statistically significant (β = 0.0250, Logit(*p*) = −1.1319 + 0.0250 × dd, *p*-value= 0.102).

Smoking status was also included in the regression model; its *p*-value for smoking was 0.078, suggesting a trend towards significance but not reaching statistical significance (β = 0.4529, Logit(*p*) = −0.8200 + 0.4529 × smoking).

The overall regression equation incorporating all variables is shown in Table 5 (Logit(*p*) = −0.6563 + 0.0283 × dd + 0.9426 × sex + 0.4529 × smoking).

The logistic model fit was evaluated using the Hosmer–Lemeshow test (stat = 0.1893, GdL = 5, *p* = 0.9992), confirming the model’s adequacy.

Moreover, a correlation analysis was performed to assess relationships between clinical and immunological markers with infection frequency. Notably, a statistically significant correlation was found between the presence of SS-A antibodies and infection rate (r = 0.291, *p* = 0.003), suggesting that specific immunological profiles may predispose patients to a higher risk of infection.

## 5. Conclusions

This study provides valuable insights into the influence of sex on the risk of infections among patients with SLE. Our findings suggest that male patients are at a significantly higher risk of infections compared to females. This is particularly relevant considering that males, although fewer in number, often present with a more severe disease course; these data are in line with the observations made by the LUMINA (LUpus in MInorities, NAture versus nurture) [14] study, a multiethnic U.S. registry including Hispanic, African American, and Caucasian patients. This study compared disease activity between males and females with SLE using specific measures of activity and accumulated damage to understand the impact of sex on the manifestations and outcomes of the disease.

Notably, the distribution of infections differed between the sexes: while upper respiratory tract infections (URTIs) were the most common in both groups, males exhibited a higher prevalence of severe infections such as pneumonia, contrasting with females who more frequently experienced urinary tract infections (UTIs). These findings are consistent with the observations made by Schwartzman-Morris and Colleagues [15], although their paper did not focus on the type and distribution of infections but rather on the clinical differences between sexes in lupus patients

Based on the data from this study, the roles of age, smoking, and disease duration in influencing infection risk in SLE patients appear nuanced and less straightforward. Unlike previous studies [5], age did not significantly differ between male and female patients, suggesting that the increased infection risk in males cannot merely be attributed to age-related factors. This highlights that other sex-specific factors, such as immunological and hormonal differences, might play a more critical role in the matter [16].

Smoking, while showing a trend toward significance, was not statistically significant in this cohort. However, smoking is generally known to compromise immune function and may still act as a compounding risk factor, especially in the presence of other vulnerabilities seen in SLE. Its role should not be entirely dismissed, as it might contribute to a cumulative effect on infection susceptibility, as seen in other reports on SLE patients.

Disease duration was shorter in males compared to females, which indicates that the higher infection risk observed in males might not be due to longer exposure to the disease.

This finding might suggest an intrinsic vulnerability to infections among males, possibly linked to differences in immune response and specific immunological profiles, such as the presence of SS-A antibodies, which were associated with higher infection rates in our analysis.

However, unlike the LUMINA study, which identified ethnicity, the lack of health insurance, and psychological factors as key contributors to disease activity, our focus was to explicitly address the impact of sex on infection risk, making our study a novel contribution to understanding how sex influences infection susceptibility in SLE.

Similarly, the work by Nusbaum et al. [6] highlighted that males with SLE often experience more severe disease manifestations, including renal involvement and cardiovascular complications, which can indirectly heighten infection risk. However, these studies did not delve into infection risk specifically, whereas our research provides direct evidence of a sex-based disparity in this domain.

Our findings suggest that the clinical management of SLE should incorporate a more tailored approach, with heightened vigilance for infections in male patients. This could lead to better prevention strategies and therapeutic interventions specifically aimed at mitigating infection risks in this vulnerable subgroup. While the findings of this study underscore the importance of sex-specific clinical management in SLE patients, additional practical guidance could enhance the applicability of these insights in real-world settings. For example, establishing recommendations on vaccination protocols tailored to each SLE patient could improve preventive care by reducing infection risks. Lastly, integrating lifestyle modification strategies, such as smoking cessation programs, may further minimize susceptibility to infections and improve overall patient outcomes. These measures could serve as valuable additions to existing management practices, ensuring a comprehensive approach to care that aligns with the unique vulnerabilities of SLE patients.

However, further research is needed to explore the underlying mechanisms of these sex-based differences, particularly focusing on immune response variations and their clinical implications.

### Limitations

This study has several limitations that should be considered when interpreting the results. Firstly, the retrospective nature of the study relies on the accuracy and completeness of medical records, which may introduce information bias, particularly in documenting infectious events and their severity. The limited number of male patients, which reflects the epidemiology of the disease, may affect the generalizability of the findings and reduce the statistical power to detect more subtle associations between risk factors and infection rates.

Additionally, all patients included in this study were Caucasian, limiting the ability to generalize these findings to other ethnic groups, who may experience different infection risks due to genetic, socioeconomic, or environmental factors. While we adjusted for some potential confounders, such as disease duration and smoking status, other unmeasured variables, including medication adherence and socioeconomic factors, may have influenced the results. This study also did not evaluate the impact of hormonal differences or detailed immunological profiles, which could further elucidate the observed sex differences in infection risk.

Finally, the study’s focus on a single center further restricts its external validity, as patient management and healthcare access can vary significantly across different settings. Prospective studies with larger, more ethnically diverse populations are needed to confirm these findings and explore the underlying mechanisms driving the observed sex-based disparities in infection risk among SLE patients.

## Figures and Tables

**Table 1:** Demographics.

	N	AGE	SD	DISEASE DURATION	SD	DISEASE SEVERITY (Mild–Moderate–Severe, %)
TOT	119	54.62	13.61	25.60	13.54	
FEMALE	107	54.3	13.62	25.30	13.62	(18; 79; 3)
MALE	12	57.25	13.56	17.98	9.47	(8; 84; 8)

**Table 2:** Comorbidities.

Comorbidity	F (*n*,%)	M (*n*,%)
Obesity	29 (27.1)	5 (41.7)
Diabetes mellitus	7 (6.6)	1 (8.3)
Hashimoto’s thyroiditis	41 (38.3)	0 (0.0)
Gastroesophageal reflux	36 (33.6)	4 (33.3)
Osteoporosis	11 (10.3)	1 (8.3)
Asthma	2 (1.9)	0 (0.0)

**Table 3:** Treatment dosage in the cohort. NS = non-significant.

Treatment	Patients, N (%)	F/M	Chi Squared (*p*-Value)
BEL	83 (69.75)	74/9	NS
AZA	19 (15.97)	18/1	NS
MMF	17 (14.29)	15/2	NS

**Table 4:** OCS dosage in the cohort. NS = non-significant.

OCS Dosage: Median, Q25, Q75	F	M	Mann–Whitney U (*p*-Value)
5.0 (5.0; 10.0)	7.5 (5.0;10.0)	5.0 (2.5;7.5)	NS

**Table 5:** Logistic regression model and Hosmer–Lemeshow test; bold: statistically significant.

Variable	Coefficient (β)	Logistic Equation	*p*-Value	Hosmer–Lemeshow Test
Disease Duration	0.025	Logit(*p*) = −1.1319 + 0.0250 × dd	0.102	
**Sex**	**0.9426**	**Logit(*p*) = −0.6061 + 0.9426 × sex**	**0.05**	
Smoking	0.4529	Logit(*p*) = −0.8200 + 0.4529 × smoking	0.078	
Overall Model		Logit(*p*) = −0.6563 + 0.0283 × dd + 1.2154 × sex + 0.3971 × smoking		stat: 0.1893, df: 5, *p* = 0.9992

## Data Availability

The raw data supporting concerning this study will be made available by the authors, without undue reservation.

## Abbreviations

SLE (systemic lupus erythematosus), OR (odds ratio), CI (confidence interval), SS-A (Anti-Ro/SS-A antibodies), TLR (Toll-like receptor), IFN-α (Interferon-alpha), ERα (Estrogen Receptor Alpha), STAT1 (Signal Transducer and Activator of Transcription 1), XCI (X-chromosome inactivation), CTI (connective tissue infection), HZV (herpes zoster virus), SARS-CoV-2 (severe acute respiratory syndrome coronavirus 2), APN (acute pyelonephritis), EBV (Epstein–Barr virus), GI (gastrointestinal), FEIA (fluorescent enzyme immunoassay), IFA (indirect immunofluorescence assay), ACR/EULAR (American College of Rheumatology/European Alliance of Associations for Rheumatology), TNF-α (Tumor Necrosis Factor-alpha), IL-1β (Interleukin-1 beta), IL-6 (Interleukin-6), IL-8 (Interleukin-8), miRNA (microRNA), anti-dsDNA (anti-double-stranded DNA antibody), C3 and C4 (Complement 3 and Complement 4 proteins), URTI (upper respiratory tract infection), UTI (urinary tract infection).
